# How, where and when to screen for porcine cytomegalovirus (PCMV) in donor pigs for xenotransplantation

**DOI:** 10.1038/s41598-022-25624-1

**Published:** 2022-12-13

**Authors:** S. Halecker, S. Hansen, L. Krabben, F. Ebner, B. Kaufer, J. Denner

**Affiliations:** 1 Institute of Virology, Free University, Berlin, Germany; 2 Institute of Immunology, Free University, Berlin, Germany

**Keywords:** Biological techniques, Immunology, Microbiology, Immunopathogenesis, Infection, Inflammation

## Abstract

Porcine cytomegalovirus (PCMV), that is actually a porcine roseolovirus (PRV), is a common herpesvirus in domestic pigs and wild boars. In xenotransplantation, PCMV/PRV has been shown to significantly reduce the survival time of pig kidneys and hearts in preclinical trials with different non-human primates. Furthermore, PCMV/PRV has been transmitted in the first pig to human heart xenotransplantation and contributed to the death of the patient. Although transmitted to the recipient, there is no evidence that PCMV/PRV can infect primate cells including human cells. PCMV/PRV is closely related to the human herpesviruses 6 and 7, and only distantly related to the human CMV (HCMV). Antiviral drugs used for the treatment of HCMV are less effective against PCMV/PRV. However, there are well described strategies to eliminate the virus from pig facilities. In order to detect the virus and to eliminate it, highly sensitive detection methods and the knowledge of how, where and when to screen the donor pigs is required. Here, a comparative testing of organs from pigs of different ages using polymerase chain reaction (PCR)-based and immunological methods was performed. Testing young piglets, PCMV/PRV was detected effectively by PCR in blood, bronchoalveolar lavage fluid, tonsils and heart. In adult animals, detection by PCR was not successful in most cases, because the virus load was below the detection limit or the virus was in its latent stage. Therefore, detection of antibodies against selected recombinant proteins corresponding to epitopes detected by nearly all infected animals in a Western blot assay is advantageous. By contrast, immunological testing is not beneficial in young animals as piglets might have PCMV/PRV-specific antibodies obtained from their infected mother via the colostrum. Using a thoughtful combination of PCR-based and immunological methods, detection of PCMV/PRV in donor pigs for xenotransplantation is feasible and a controlled elimination of the virus by early weaning or other methods is possible.

## Introduction

During the first pig heart xenotransplantation into a human patient at the University of Maryland in Baltimore, the porcine cytomegalovirus (PCMV) that is actually a porcine roseolovirus (PRV) (PCMV/PRV) was transmitted to the patient^1^. This virus is known to significantly reduce the survival time of pig xenotransplants in non-human primates. In the very first preclinical xenotransplantations of pig thymokidneys into baboons an activation of the PCMV/PRV was observed^2^. The animals showed a consumptive coagulopathy, a combination of thrombocytopenia, decreasing fibrinogen levels and uncontrollable bleeding^3^. These symptoms also had been seen in the Baltimore patient. A significant reduction of the survival time of pig kidneys in baboons^4^ and cynomolgus monkeys^5^ was reported. The same was demonstrated for pig hearts orthotopically transplanted into baboons: hearts from α1,3-galactosyltransferase-knockout (GTKO) pigs that express human membrane cofactor protein (CD46) and human thrombomodulin (hTM) survived no longer than 30 days when infected with PCMV/PRV, whereas baboons that received PCMV/PRV-free organs survived for 182 and 195 days^6^.

PCMV/PRV belongs to the genus *Roseolovirus*, and it is closely related to the human herpes viruses (HHV)-6A, -6B and -7^7^. It is only distantly related to the human cytomegalovirus (HCMV), which belongs to the genus *Cytomegalovirus*. This data and further details on PCMV/PRV have been summarized in several reviews^8–10^.

PCMV/PRV is widely distributed in pig populations world-wide^11–13^. In a random sampling from a German slaughterhouse nearly all animals were tested positive^14^. PCMV/PRV was also found in wild boars^15–17^. PCMV/PVR is mostly acquired early in life and infection results in seroconversion and life-long latent infection^11^. An active infection with PCMV/PRV causes fatal systemic failure in piglets less than 3 weeks of age. The clinical symptoms of infected piglets include pneumonia, inclusion body rhinitis, and a high mortality rate. PCMV/PRV-infected sows are prone to abortion with pathological changes including edema in the heart, lungs, lymph nodes, and mesocolon^11^. Viral excretions were observed in young infected animals, mainly by nasal secretions. Virus excretion usually began when piglets were between 3 and 6 weeks of age and reached a maximum between 5 and 8 weeks; it was usually no longer detectable at 11-12 weeks^18^. Antibodies of maternal origin declined in titre between 2–3 and 5–6 weeks. Later the majority of piglets showed seroconversion as a result of virus infection^17^. In pigs, the epitheliochorial placenta does not allow the passage of both antibodies and immune cells from sow to fetus, so the newborn pig survival depends on the intake of maternal derived antibodies within colostrum and milk. The colostrum contains maternal IgG, IgM, and IgA, which pass through the enterocytes into the bloodstream due to a complete bowel permeability. Within 24 h, IgGs reach a serum concentration comparable to its mother^19^. The colostrum also contains T and B lymphocytes, which can also pass into neonate’s blood.

Based on all available data, PCMV transmission happens from mother sows to their piglets after birth and transmission via placenta was observed only under experimental settings^20^, but not under natural conditions^18,21,22^. When adult animals from a high-hygienic herd were evaluated, older animals in particular showed positivity, and within a given litter not all animals were PCMV positive. These data fit with spread through the herd by horizontal transmission, not in utero infection^23^. One study demonstrating generation of PCMV/PRV-negative piglets from virus positive mothers by early weaning^24^ shows in addition that the risk of virus transmission by the colostrum, which was still given to these animals, was zero.

In the blood of the baboons that received an orthotopic PCMV/PRV-positive heart high virus loads were measured^6^. The virus, obviously, replicated unrestrained in the transplanted pig heart, which was not any more under the control of the immune system of the pig. Cells producing PCMV/PRV proteins or possibly infectious virus were found in many organs of the baboon recipient^25^. These cells were thought to be disseminated pig cells derived from the transplant. Until now, there is no evidence that PCMV/PRV is able to infect human cells and cells of other primates^26^. It is still unknown how the virus contributes to the symptoms observed in non-human primates and in the Baltimore patient. An examination of the baboons after the orthotopic transplantation of PCMV/PRV-positive pig hearts showed increased levels of interleukin 6 (IL-6), tumour necrosis factor α (TNFα) and high levels of tissue plasminogen activator-plasminogen-activator-inhibitor (tPA-PAI-1) complexes^6^. These findings suggest a complete loss of the pro-fibrinolytic properties of the endothelial cells. It seems that viral proteins directly interact with endothelial and immune cells of the recipient eventually causing multiorgan failure.

Like all herpes viruses, PCMV/PRV is able to establish latency, which makes it difficult to detect the virus. During latency the viral DNA is stably maintained in the nucleus of the infected cells and the protein expression in latently infected cells is decreased^27,28^. In this situation, highly sensitive detection methods are needed and the knowledge what and how to test. The absence of a reliable method and test strategy was the reason why PCMV/PRV was not detected in the donor pig used for the Baltimore patient. Different PCR-based detection methods have been developed in order to detect the viral DNA genome^2,23,29^. In latency, however, the virus load is often under the detection limit of the applied PCR method. Hence, immunological assays have been developed to screen for antibodies against PCMV as indirect sign of previous virus infection. These assays were based on immunofluorescence using virus-infected cells^18^ or Western blot assays using recombinant fragments of the viral glycoprotein B (gB), which effectively identified PCMV-positive animals even in the case of a negative PCR result^14^.

Here, we investigated blood and different tissues from juvenile and adult pigs in order to identify material with the highest probability to detect viral DNA. Additionally, the PCMV/PRV load in the pig heart as a relevant xenotransplant was analysed. In parallel, we screened for antibodies using a Western blot assay based on a recombinant fragment of the gB of PCMV/PRV.

## Results

### Detection of PCMV/PRV in the blood and different tissues of juvenile pigs

**Figure 1:**
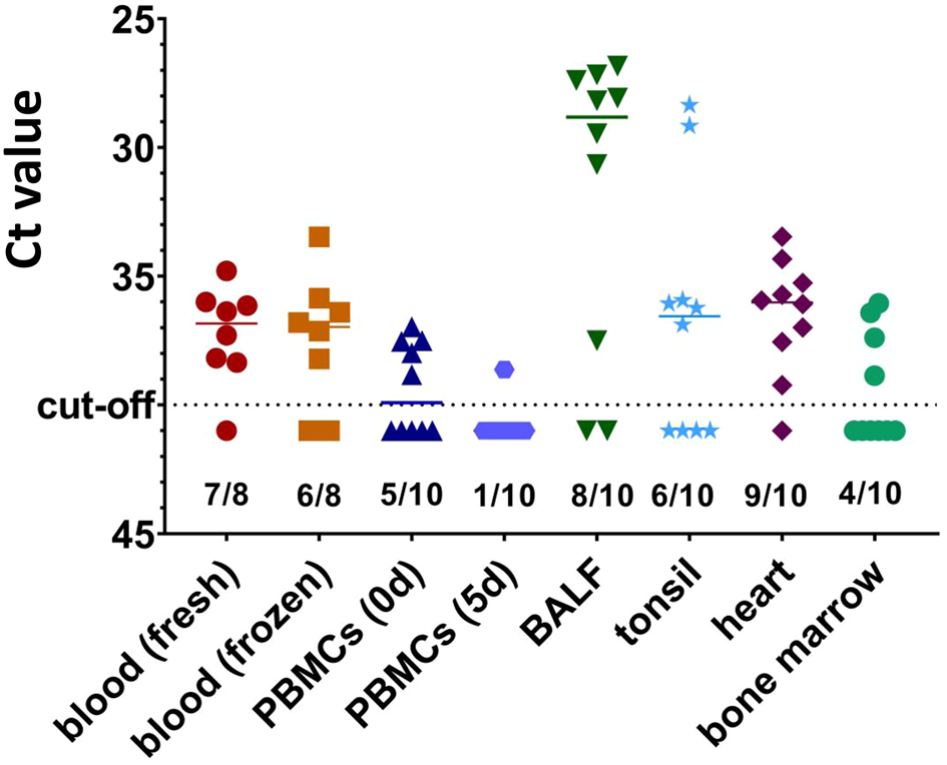
Detection of PCMV/PRV in the blood and different tissues of ten 9 weeks old piglets by real-time PCR. The number of positive/tested animals is indicated. PBMCs, peripheral blood mononuclear cells; PBMCs were tested at day 0 (0d) and after 5 days (5d) cultivation, BALF, bronchoalveolar lavage fluid.

Ten 9-weeks old piglets were tested for PCMV/PRV by real-time PCR. The following tissues were analysed: Peripheral blood mononuclear cells (PBMCs), purified from blood, PBMCs cultivated in culture medium for 5 days, cells from bronchoalveolar lavage fluid (BALF), soft palate tonsils, heart and bone marrow. In addition, full blood was tested fresh and after one freeze-thawing process. DNA was isolated and a real-time PCR (named PCMV 1, Table 1) was performed as described in Methods. In fresh blood samples seven out of eight animals (blood samples from two animals were not available) showed a cycle threshold (Ct) value between 35 and 38 (Ct cut-off 40); in frozen blood six out of eight piglets were PCMV/PRV-positive (Fig. 1, Supplementary Table 1). Isolated fresh PBMCs were positive in five out of ten animals. When the PBMCs were cultured for 5 days in fetal calf serum-containing culture medium, only one out of ten samples was positive. BALF cells from eight out of ten animals were tested positive for PCMV/PRV, seven of the positive ones showed a low Ct value (< 31), indicating a high virus load. Tonsil tissue revealed six out of ten positive animals, two of the positive had also a low Ct value. In heart samples nine out of ten piglets were positive; bone marrow showed four out of ten positive animals.

**Table 1:** Primers and probes used in this study.

PCR assay	Primer/probe	Sequence 5ʹ-3ʹ	Amplicon (base pair)	References
PCMV 1	PCMV-Fwd	ACT TCG TCG CAG CTC ATC TGA	63	^2,17,30^*
	PCMV -Rev	GTT CTG GGA TTC CGA GGT TG		
	PCMV -Probe	6FAM-CAG GGC GGC GGT CGA GCT C-BHQ		
PCMV 2	Fr-t (nt279-299)** Rr-t (nt373-354)** Probe (nt331-350)**	AAT GCG TTT TAC AAC TTC ACG CTG AGC ATG TCC CGC CCT AT 6FAM-CTCTAGCGGCGTCCATCACC-BHQ		^31^
pGAPDH	pGAPDH-Fwd	ACA TGG CCT CCA AGG AGT AAG A	106	^32^
	pGAPDH-Rev	GAT CGA GTT GGG GCT GTG ACT		
	pGAPDH-Probe	HEX-CCA CCA ACC CCA GCA AGA GCA CGC-BHQ1		

*The PCMV real-time PCR was modified and performed as duplex PCR.

**nt, nucleotide, GenBank AJ222640.

*PCR* polymerase chain reaction, *PCMV* porcine cytomegalovirus, *pGAPDH* porcine glyceraldehyde-3-phosphate dehydrogenase, *Fwd* forward primer, *Rev* reverse primer, *nt* nucleotide, *6FAM* 6-Carboxyfluorescein, *BHQ* black hole quencher, *HEX* Hexachloro-fluorescein.

This data show that in BALF cells the highest virus load was found. However, obtaining BALF from a pig designated for xenotransplantation is very laborious and may be associated with certain risks for the donor animal and the transplant, in this case anesthesia and short term neutropenia. The same would be true for the tonsil samples, heart biopsies (especially in the case of a heart transplantation) and bone marrow. To note, all ten animals are infected, as at least three samples of each animal were positive for PCMV/PRV (Supplementary Table 1).

### Detection of PCMV/PRV in blood and PBMCs of adult pigs

Three animals were monitored for PCMV/PRV from week 12 (or 14) until week 41 (or 43) using the same real-time PCR method as used for the tissues and blood from the piglets (PCMV 1, Table 2). Fresh blood samples were positive until week 17 (pig U) or week 12 (pig V); later both animals showed no Ct values as pig W (Table 2). Fresh blood, frozen blood, fresh PBMCs, PBMCs after five days cultivation with or without PHA, from 41 or 43 weeks old animals, showed negative results for PCMV/PRV (no Ct) (Supplementary Table 2), suggesting that there is no replicating virus, replicating virus below the detection limit or the virus went to latency. Increasing the number of cells used for DNA isolation from 1 × 10^6^ to 3 × 10^6^ in order to increase the probability that an infected cell is among the tested ones or using an DNA isolation kit from another company which eluted the DNA at a higher concentration, did not change the result (Supplementary Table 2).

**Table 2:** Results of the real-time PCR testing of blood and the Western blot analysis or three pigs at four different time points corresponding to the age of the animals.

Animal	Gender	Real-time PCR results (Ct)				Western blot assay	
		14 weeks	17 weeks	19 weeks	43 weeks	14 weeks	43 weeks
Pig U	female	**35.3**	**39.0**	*No Ct*	*No Ct*	**Positive**	**Positive**
		12 weeks	15 weeks	17 weeks	41 weeks	12 weeks	41 weeks
Pig V	female	**37.6**	*No Ct*	*No Ct*	*No Ct*	**Positive**	**Positive**
Pig W	female	n.s	n.s	n.s	*No Ct*	n.s	**Positive**

n.s.—no sample available. *PCR* polymerase chain reaction, *Ct* cycle threshold.

Positive values are in bold and negative values (no ct) in italics.

Furthermore, another PCR system (named PCMV 2, Table 1) was used, which was developed previously and found very sensitive^31^. Using this PCR, pigs U, V and W were also found negative (Supplementary Table 3). A log_10_ dilutional series of PCMV-positive porcine DNA was comparatively tested with the PCMV 1 and the PCMV 2 PCR assays. The results showed that the PCMV 1 assay is one dilution level of a log_10_ dilutional series more sensitive than the PCMV 2 assay (Supplementary Table 3). The PCMV 1 also revealed lower Ct values than PCMV 2 for identical samples indicating to be more sensitive than PCMV 2 assay.

### Screening for antibodies against PCMV/PRV

**Figure 2:**
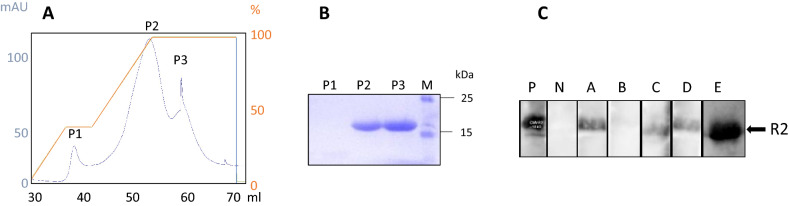
(**A**) HisTrap column elution profile of the recombinant R2 fragment of gB of PCMV/PRV, (**B**) SDS-PAGE of the purified R2 fragment. P2 and P3 correspond to the peak fractions shown in (**A**), (**C**) Result of the Western blot analysis of sera from adult pigs A, B, C, D and E. A to E pigs were around 4 months old. P, positive control, N, negative control. Serum dilution 1:300, goat anti-pig horse reddish peroxidase was used for detection. Blots were cut prior to hybridization with antibodies during blotting, each stripe was incubated with an individual serum.

To screen for antibodies against PCMV/PVR as indirect evidence of a virus infection, a Western blot assay as described previously^14^ was performed. Two fragments of the glycoprotein B (gB) of PCMV/PRV, a N-terminal R1 and a C-terminal R2 (sequences 539–929 and 2771–16,663 of the gB of PCMV, Acc. No: AF268039), had been used. However, many tests in the past have shown that the vast majority of the infected animals reacted with the R2 fragment^14^. Therefore, we concentrated on fragment R2. The recombinant protein was produced in *E. coli*, purified by affinity chromatography (Fig. 2A), characterized by SDS-PAGE (Fig. 2B) and used as antigen in Western blot assays (Fig. 2C). Sera from five adult pigs (designated A to E) were examined. All animals had been tested PCMV-negative in the PCR assay (not shown). All but one of the adult animals A to E reacted with the R2 band on the blot membrane (Fig. 2C).

**Figure 3:**
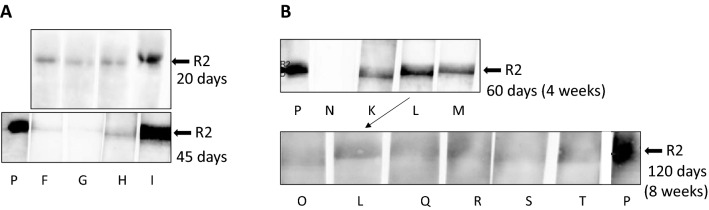
Detection of antibodies against the recombinant R2 fragment of the glycoprotein B in young animals. (**A**) Detection of antibodies against the R2 protein in the sera of four animals (F, G, H, I) after 20 days and after 45 days. (**B**) Detection of antibodies against the R2 fragment in the sera of eight animals (K, L, M, O, Q, R, S, T) from the same litter at day 60 and day 120. The serum from animal L was tested on both days. Animals O, L, Q, R and S, were male, T was a female. P, positive control, N, negative control. Serum dilution 1:300, goat anti-pig horse reddish peroxidase was used for detection. Blots were cut prior to hybridization with antibodies during blotting, each stripe was incubated with an individual serum.

This Western blot assay was also used to screen young piglets. Sera from piglets designated F to I, which had been tested negative by PCR (not shown), gave a weak positive reaction in the Western blot assay at day 20 after birth (Fig. 3A, upper panel). We proposed that this result may be due to maternal antibodies derived from the colostrum that the animals received from their infected mother sow. When we tested the same animals again 45 days after birth, a rapid decline of the antibody concentration in two of the four animals was observed. Only in the serum of pig I the antibody titer increased, indicating that this animal is infected and reacts with own antibodies against the infection (Fig. 3A, lower panel).

To confirm this result, a Western blot analyses was performed with sera from eight animals from a single litter at day 60 and day 120 (Fig. 3B). A significant decline of the antibody titer was observed, indicating that the maternal antibodies transmitted by the colostrum disappeared.

**Figure 4:**
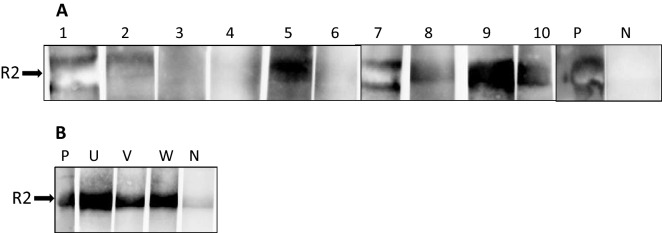
Result of the Western blot analysis using the recombinant R2 fragment of the glycoprotein B of PCMV/PRV. (**A**) nine weeks old animals (number 1 to 10) with positive PCR results in at least one tissue (Fig. 1, Supplementary Table 1) and (**B**) the pigs U, V, and W, 43 or 41 weeks old and which were PCR-negative (Table 2). P, positive control, N, negative control. Serum dilution 1:300, goat anti-pig horse reddish peroxidase was used for detection. Blots were cut prior to hybridization with antibodies during blotting, each stripe was incubated with an individual serum.

When sera from the ten nine weeks old piglets (animals 1–10), which were found positive in at least in three tissues when tested by PCR (Fig. 1, Supplementary Table 1) were analysed in the Western blot assay, seven of the animals were positive (Fig. 4A). Four of them showed a very strong reaction. Since antibodies from the colostrum disappeared at latest 8 weeks after birth (Fig. 3A,B), this strong reaction can only be explained by a de novo antibody production in response to the virus infection.

Sera from adult pigs U, V, and W, which were older than 41 weeks, and which were tested negative for PCMV/PVR in real-time PCR from week 17 on (Table 2) were all strongly positive in the Western blot assay (Fig. 4B). Hence, the animals U, V, and W were infected with PCMV/PVR despite the negative PCR result.

## Discussion

In the first transplantation of a pig heart into a human patient a pig virus, PCMV/PRV, was transmitted to the patient^1^. This underlines the requirement to pay more attention to the aspects of virus safety in xenotransplantation. Here, we demonstrate how difficult it is to detect this virus, especially in his latent state. But we also demonstrate that using a combination of PCR-based and immunological assays, it is possible to detect the virus and using these methods also to eliminate PCMV/PRV.

There are three main aspects of testing: how, when and where. First, how to test: The modified real-time PCR developed by Mueller et al.^2^ is a sensitive and specific tool to detect the virus. We modified the method performing a duplex real-time PCR that detect the PCMV/PRV DNA and simultaneously an internal control, the cellular gene porcine glycerinaldehyd-3-phosphat-dehydrogenase (pGAPDH)^30^. The pGAPDH indicates a successful extraction of DNA and a correct performance of the PCR assay. In addition, we developed several new real-time PCRs and nested PCRs and analyzed Göttingen minipgs (GöMP) which are potential donor pigs for clinical pig islet cell transplantations in Germany^31,33^. The sensitivity of these tests was down to 2–5 DNA copies. We previously compared a real-time PCR, which was newly developed (here called PCMV 2), with a nested PCR and a real-time PCR performed in an American commercial laboratory^23^. Testing 10 GöMP, our real-time PCR detected PCMV/PRV in 4 sera, while our nested PCR detected the virus in 3 sera, and the commercial real-time PCR detected no virus in all pigs. Hence, it is not only important to have a PCR or a real-time PCR method that detects the virus in principle but also to know its sensitivity, e.g., how many virus particles can be detected by this approach. When we compared the modified duplex real-time PCR using primers and probe developed by Mueller et al.^2^ (here called PCMV 1), with the duplex real-time PCR PCMV 2, we found that their sensitivity is approximately the same (Supplementary Fig. 3).

Second, when to test: Our results show that in young piglets the virus is detected by real-time PCR easily, but not by antibody detection: If the mother sow is infected there are maternal antibodies from colostrum in the blood (Fig. 3). Approximately after 45 days the titer started to decline and after 120 days the maternal antibodies almost completely disappeared. This result correlate well with other findings^18^. In adult pigs, it is nearly impossible to detect the virus in blood samples by PCR, nested PCR or real-time PCR if the virus is in its latent state. However, our Western blot assay demonstrated that the pigs had developed antibodies against PCMV/PRV indicating an infection (Table 2, Fig. 4B). Due to the latent state of the virus a once infected pig has to be considered PCMV/PRV-positive for its life time.

Third, where to test: For diagnostic purposes blood is often used. In the present study, PCMV/PRV was detected by real-time PCR in the blood from seven out of eight young animals (Fig. 1), but in none of the adult animals (Table 2), similar to former results^33^. It is important to note, that all ten animals tested were PCMV/PRV-positive (Supplementary Table 1). However, the tissues, which were found positive differed from animal to animal (Fig. 1 and Supplementary Table 1). This shows that the distribution of the virus in different animals (which were housed together) varied. In a previous study PCMV/PRV was found mainly in the kidney, but also in the pancreas, liver and spleen of a genetically modified pig^25^. In parallel, the organs from three related pigs of the same age were screened: In two animals the highest virus load was found in the nose, in one animal in the spleen. Lower amounts were found in the heart and kidney. Altogether, this data shows, that the distribution of PCMV/PRV is very complex and difficult to predict^25^. When tissue samples of these animals were tested by immunohistochemistry using specific antibodies no PCMV/PRV was detected in the liver, spleen, kidney, heart, lung and lymph nodes. This indicates the low sensitivity of immunohistochemistry and the low or more likely the absent expression of viral proteins in the latent state of virus infection^25^.

In previous publications PCMV/PRV was found in infected embryos mainly in leptomeningeal cells, hepatic sinusoidal cells, peritoneal macrophages, periosteal cells and occasional alveolar cells, but the placenta did not appear to be a primary site of viral replication^21^. If seropositive sows were infected again with the virus a superinfection occurred that in a few cases resulted in fetal deaths and congenitally infected pigs. However, a superinfection cannot be categorically distinguished from a reactivation of the virus. PCMV/PRV was found in the capillary endothelium and alveolar macrophages from the lungs of some fetuses^22^. Virus was also isolated from the nasal mucosa. Some fetuses yielded virus from the spleen and central nervous system, the endothelial cells of the cerebrum showed positive immunofluorescence^22^.

Other authors investigated human decay accelerating factor (CD55) transgenic pig herds being bred for xenotransplantation, but kept under conventional farm conditions. They identified PCMV/PRV in a wide range of organs including potential xenotransplants (liver, kidney and heart). The spleen was PCMV/PRV DNA positive in all infected pigs^34^. In another study five pig tissues (lung, liver, salivary gland, kidney, and gut) derived from multiple animals from a well-characterized herd of inbred miniature swine were tested for PCMV/PRV using a PCR. Lung, liver, salivary gland, and kidney were always positive, the gut tissue was consistently PCR negative^35^.

In order to test whether low-invasive or non-invasive methods can be applied which would allow performing regular follow-ups and further breeding of the animals, we tested sera, ear biopsies, as well as oral and anal swabs collected from ten 10-day-old Aachen minipigs (AaMP) by a sensitive nested PCR as well as by uniplex and duplex real-time PCR^36^. PCMV/PRV DNA was detected most frequently in oral and anal swabs. To note, comparison of duplex and uniplex real-time PCR systems demonstrated a higher sensitivity of uniplex real-time PCR when the copy numbers of the target genes were low (less 200). Please note, the piglets were 10 days old.

We were surprised that only one out of ten animals was identified PCMV/PRV-positive when gradient-purified PBMCs were cultured for 5 days (Fig. 1A). In contrast, screening of fresh PBMCs of these animals yielded five out of ten animals positive. In a previous study cultivation for five or seven days even resulted in an activation of the virus and a higher probability to detect the virus^25^. Obviously, in the experiment here the virus-positive cells died during cultivation or adherent PCMV/PRV-positive macrophages were lost when testing.

When pigs from a multiplier unit operated by Spring Point Project were screened for PCMV/PRV, the virus was detected in the spleen of all tested six animals, isolated islet cells from three of the six animals were positive and two of these three cases were even positive in cultured islets^23^.

Recently, we demonstrated that there is a cross-reactivity between antibodies directed against PCMV/PRV and HHV-6 and that sera from HHV-6-positive humans react in our Western blot assay^37^. It will be interesting to learn, whether the antibodies against HHV-6 present nearly in each human individual will have an impact on PCMV/PRV transmission, either reducing the virus transmission or even enhancing it for example by enhancing antibodies, i.e., antibody-dependent enhancement (ADE) or by harmful antibodies directed against the PCMV antigens expressed in the pig transplant. At the moment there is no information about the presence of antibodies against HHV-6 and PCMV in the Baltimore patient, but it was shown that the patient was HHV-6 positive^1^. It is important to note that our Western blot assay using the two recombinant protein fragments of the gB of PCMV/PRV can only be used for the detection of an PCMV/PRV transmission when the patient is HHV-6 negative. However, if PCMV/PVR replicates well and the virus load is high, the viral DNA can be detected easily by PCR as was seen in the Baltimore patient.

There is another interesting aspect learned from the transmission of PCMV/PRV into non-human primate recipients and into the Baltimore patient. Numerous publications described a systemic inflammation in xenotransplant recipients (SIXR)^38–40^ and a detrimental role of inflammation in pig orthotopic heart xenotransplantation^41^. SIXR is characterized by activated innate immune cells expressing tissue factor (TF). C-reactive protein, fibrinogen and interleukin 6 (IL-6) are extremely increased. The increased C-reactive protein levels are observed prior the development of consumptive coagulopathy^38–41^. Some of these features were also found in the patient in Baltimore and in baboons which received a PCMV-positive pig transplant, for example increased levels of IL-6 and TNFα and disruption of coagulation^2–6^. In vitro, an activation of the porcine TF in porcine aortic endothelial cells by PCMV/PRV infection was observed^3^. Therefore, it will be of great importance to dissect the effects of undetected PCMV/PRV infection (not tested or tested with the suboptimal method) and reaction against the xenotransplant.

An excellent testing is also important for monitoring antiviral treatment. Antiviral drugs which are successfully used to treat HCMV infections in humans such as ganciclovir, valacyclovir, cidofovir have also been show the be to some extend effective against PCMV/PRV in vitro^42^. However, the situation in vivo is different^43^. PCMV viral loads were unaltered by ganciclovir treatment. Only cidofovir and foscarnet might have therapeutic efficacy for PCMV in vivo in achievable concentrations, although these agents often carry significant toxicity in transplant recipients. Ganciclovir, valacyclovir and cidofovir were used to treat the Baltimore patient^1^. After the first application of cidofovir at day 43, the PCMV/PRV load in the patient continued to increase, but after the second application (day 49) it seemed to decrease. However, at day 43 the patient received also an injection of human IgG. This preparation contained most likely antibodies against HHV-6 due to its high seroprevalence of > 90% in the human population^44^. We have recently shown^37^ that antibodies against HHV-6 are cross-reactive against PCMV/PRV. Therefore, these antibodies could be the reason for the decrease in virus load.

Although high PCMV/PRV virus load was detected in the patient and some of the clinical features resemble the ones observed in the baboons with PCMV/PRV-positive hearts^1,6^, it remains unclear as to which part the PCMV/PRV contributed to the death of the patient, who was already very ill before transplantation^45^. One of the factors, which may have been also contributed is the application of human IgG^46^. As was found later, this preparation contained antibodies against pig tissues.

It would be useful to have an effective drug against PCMV or even a vaccine able to prevent virus transmission. But the virus transmission can be prevented by easy methods such as early weaning^24^. Since under natural conditions no transmission of the virus via the placenta was observed^18,23^, cesarean delivery^34^ or embryo transfer are not necessary to remove the virus. Even colostrum deprivation does not seem to be required, since early weaning alone was successful^24^. However, isolated breeding and housing of the animals + is required to avoid a re-entry of the virus into a PCMV/PRV-free facility. A re-entry of the virus may also happen by cloning or somatic cell nuclear transfer SCNT using oocytes from slaughterhouses, which normally are PCMV/PRV-positive^47^.

**Figure 5:**
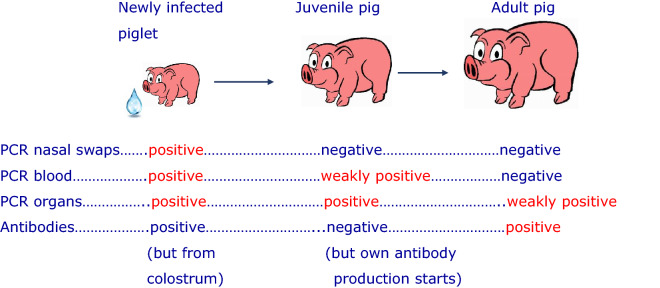
Detection strategies for PCMV/PRV in pigs of different age and infection status. The results of a given method marked in red represent a reliable result when used at the correct time with the correct test material. If possible, PCR and antibody testing should be performed simultaneously and the tests should be repeated, especially in the case of newborn and juvenile animals.

Taken together, it is possible to detect and eliminate PCMV to guaranty safe future clinical xenotransplantations using effective methods (Fig. 5). There is no need to re-open the discussion that xenotransplantation may introduce new zoonotic viruses into the human population^43^.

## Methods

### Animals, blood and other tissues

For the molecular biology detection of PCMV, samples from ten Large White x Landrace crossbred pigs (9 males, 1 female) were repurposed from an unrelated animal experiment. Piglets were purchased from a commercial provider with the age of 5 weeks and maintained in two separated groups at the animal facility at the Free University Berlin. At the age of nine weeks, lithium-heparin (LH) blood and serum were sampled by heart puncture of sedated animals (Ursotamin; Serumwerk Bernburg AF 20 mg/kg body weight (BW), Azaperon; Janssen-Cilag GmbH 2 mg/kg BW and Xylavet; CP-Pharma Handelsgesellschaft mbH 36 mg/kg BW) before euthanizing the pigs by intracardial injection of T61 (10 mg/kg BW; Intervet, Unterschleißheim, Germany). A portion of LH blood was processed immediately, while the other portion was frozen away at − 20 °C. Serum samples were stored at − 20 °C.

Bronchoalveolar lavage (BAL) was performed on isolated stanched lungs by inserting a tracheal tube through an incision in the right bronchus and flushing the lung lobe with 250 mL phosphate buffered saline (PBS) containing 2 mM EDTA. BAL fluid (BALF) was filtered (70 µm cell strainer) and BALF cells were pelleted and counted before being stored at − 20 °C. A sample of heart muscle tissue was taken from the cardiac apex. Tonsils of the soft palate were sampled as major pharyngeal mucosal-associated lymphoid tissue. Tonsillar tissue and heart tissue samples were shock-frozen in liquid nitrogen after their removal and stored at − 80 °C until further processing. For isolating bone marrow cells five posterior ribs were extracted per pig and stored over night at 4 °C. The following day, muscular and connective tissues were removed and the outer surface was treated with 70% ethanol before cutting the ribs in 2 cm bone fragments and flushing out the bone marrow with RPMI-1640 (PAN-Biotech GmbH) supplemented with 2 mM EDTA. Bone marrow cells were pelleted by centrifugation and resuspended with ice cold ammonium-chloride-potassium lysing buffer for 2 min at room temperature to lyse red blood cells before cell numbers were determined. Bone marrow cells were frozen at − 80 °C.

Sera from young piglets at the age of 20, 45 and 60 days were obtained from a pig facility at the Chair of Livestock Biotechnology, Technical University Munich, School of Life Sciences Weihenstephan, Germany) (courtesy of Dr. Konrad Fischer). Blood and PBMCs from adult pigs at different time points from 12 to 43 weeks were obtained from the Teaching and experimental farm of the Faculty of Veterinary Medicine of the Ludwig Maximilians University Munich, Germany) (courtesy of Dr. Barbara Keßler and Prof. Eckard Wolf).

### Preparation and cultivation of peripheral blood mononuclear cells (PBMCs)

To separate the PBMCs from the fresh EDTA or LH blood samples of the pigs, a density gradient medium (Pancoll human, density: 1.077 g/mL, PAN Biotech GmbH, Aidenbach, Germany) were poured in a centrifugation tube and carefully overlayed with EDTA and LH blood, respectively. The blood-medium mix was centrifugated for 25 min at 900 × *g*, the PBMCs were separated and washed twice with PBS by centrifugation at 300 × *g* for 10 min. For cultivation, 1 × 10^6^ PBMCs per sample were seeded in 12-well plates (Sarstedt AG & Co. KG, Nümbrecht, Germany), solubilized in 2 mL Roswell Park Memorial Institute medium (RPMI-1640, PAN-Biotech GmbH, Aidenbach, Germany) and harvested after 5 days at 37 °C, 5% CO_2_. In a parallel experiment, phytohemagglutinin (PHA-L) (Invitrogen, Whaltham, MA, USA) was added at a concentration of 2.5 µg/mL to the PBMC culture.

### DNA extraction

DNA of most samples were purified by using the DNeasy Blood & Tissue kit (Qiagen, Hilden, Germany). According to the results of pre-tests (data not shown here), a volume of 80 µL of fresh and frozen blood were used for the nucleic acid extraction and further processing was carried out according to the manufacturer's recommendations. For the nucleic acid extraction of the PBMC and BALF samples, 1 × 10^6^ cells were used. The DNA extraction of the organs (tonsils, heart, bone marrow) was based on 20 mg of each tissue. All samples were eluted in 200 µL elution buffer. In addition, two alternative methods of DNA isolation were performed: To confirm the negative results, a higher number of PBMCs (3 × 10^6^ cells) (modification 1, Supplementary Table 2) and the innuPREP Virus DNA/ RNA Kit (Analytik Jena, Jena, Germany) (modification 2) were used according to the manufacturer´s instructions. RNA/DNA from 3 × 10^6^ cells was eluated in 30 µL nuclease-free water. The samples were stored at − 20 °C until further processing.

### Real-time polymerase chain reaction (PCR)

The detection of PCMV/PRV was performed by two real-time PCR assays (PCMV 1 and PCMV 2) based on TaqMan technology as described previously^31,30^. All experiments were performed with the SensiFAST Probe No-ROX kit (Meridian Bioscience, Cincinnati, OH, USA) at the qPCR cycler qTOWER3 G (Analytik Jena, Jena, Germany). All assays were performed as duplex real-time PCR using the reference gene porcine pGAPDH and using a specific primer–probe mixture (Table 2). A reaction volume of 20 µL was prepared containing 1.8 µL of PCMV-FAM mix and 1.8 µL of pGAPDH-HEX mix as internal control and 4.0 µL of extracted DNA. The reaction for the PCMV 1 PCR was carried out for 2 min at 50 °C for activation, 10 min at 95 °C followed by 45 cycles comprising 15 s at 95 °C for denaturation and 60 s at 60 °C for annealing and elongation. For the PCMV 2 PCR the following conditions for amplification were used: denaturation at 95 °C for 5 min and 45 cycles of amplification with denaturation at 95 °C for 10 s, annealing at 59 °C for 20 s and extension at 60 °C for 25 s.

### Protein expression and purification

The R2 fragment of the gB of PCMV/PRV was purified as described^14^. In brief, 500 mL *E. coli* BL21 cells containing the pET16b expression vector encoding PCMV-R2 were induced with 1 mM isopropyl-β-D-thiogalactopyranosid (IPTG) (Roth, Karlsruhe, Germany) at an OD_600_ of 0.7 and cultivated at 37 °C for 2 h. Cells were harvested, centrifuged, resupended in PBS and centrifuged again. 2 g wet cells were dissolved in 10 mL 8 M urea, 0.5 M NaCl, 15 mM imidazole, 20 mM Tris pH 7.5. After a 20 min spin at 50,000 × *g* the supernatant is applied to a 1 mL HisTrap HP column installed on a Äkta Prime Plus system (both GE Healthcare, Chicago, Illinois, USA) using the HisTrap affinity application template with the following buffers: (1) 6 M urea, 0.5 M NaCl, 15 mM imidazole, 20 mM Tris pH 7.5; (2) 6 M urea, 0.5 M NaCl, 500 mM imidazole, 20 mM Tris pH 7.5. PCMV-R2 eluted in two peaks as was shown by polyacrylamide-gel electrophoresis. Both fractions were used for PCMV antibody detection.

### Western blot assay

For the detection of antibodies against PCMV/PRV, the Western Blot assay designed by Plotzki et al.^14^ was re-established. The purified R2 protein was characterized by a sodium dodecylsulfate polyacrylamide gel electrophoresis (SDS-PAGE). The protein was dissolved in sample buffer (375 mM Tris–HCl, 60% glycerol, 12% SDS, 0.6 M DTT, 0.06% bromophenol blue) and denatured for 5 min at 95 °C prior to electrophoresis. The SDS PAGE was run in a Mini-Protean Tetra Vertical Electrophoresis Cell (Bio-Rad Laboratories, Incs., Hercules, CA, USA) using a 12% polyacrylamide gel and the PageRuler prestained protein ladder (Thermo Fisher Scientific, Waltham, USA). The protein was transferred for 100 min to a Polyvinylidene fluoride membrane (ROTI PVDF, 8989.1, Roth, Karlsruhe, Germany) by electroblotting (100 mA) using the electroblotting device of peqlab Biotechnologie GmbH. Blots were cut prior to hybridization with antibodies during blotting, each stripe was incubated with an individual serum. After electroblotting the membrane was blocked for 1 h at 4 °C in 5% non-fat dry milk (Roth, Karlsruhe, Germany) in PBS with 0.05% Tween 20 (Roth, Karlsruhe, Germany) (blocking buffer). Membrane was cut into strips and incubated with sera diluted 1:300 in blocking buffer at 4 °C overnight. Afterwards, washing was performed with 0.05% PBS-T three times for 10 min each. Polyclonal goat anti-pig immunoglobulin G (IgG) Fc Secondary Antibody HRP (Invitrogen by Thermo Fisher Scientific, Waltham, USA) was diluted 1:20.000 in blocking buffer and the strips were incubated for 1 h at room temperature, followed by three washing steps for 10 min each. Detection of the signal following incubation with the ECL Western Blotting Substrate (Cytiva, Amersham) was done on the imaging device FUSION-SL 3500 WL (peqlab Biotechnologie GmbH).

### Ethics statement

Animals were cared for in accordance with the principles outlined in the European Convention for the Protection of Vertebrate Animals used for Experimental and other Scientific Purposes and the German Animal Welfare Law. All methods are reported in accordance with ARRIVE guidelines. Ethical approval was obtained from the State Office of Health and Social Affairs Berlin, Germany (Landesamt für Gesundheit und Soziales Berlin, Germany) for sampling tissues from healthy pigs (Regulation Number T0002/17) and for experimental procedures (Approval Number G0278/18). Animals shown in Fig. 1 were sedated using Ursotamin and Xylavet and euthanizing by intracardial injection of T61 as described in Material and methods. It is important to note that all materials were obtained from animals being in another experiment.

## Supplementary Information

Supplementary Information.

## Data Availability

The dataset generated and analyzed during the current study are all available in this manuscript including the Supplementary Tables and Supplementary Information files showing full-length gels and blots.
