# Supplementary material for: How, where and when to screen for porcine cytomegalovirus (PCMV) in donor pigs for xenotransplantation
Source: Sci Rep. 2022 Dec 13;12:21545. doi: 10.1038/s41598-022-25624-1 (PMC9747970; doi:10.1038/s41598-022-25624-1)
Supplement: Supplementary file 1 — Supplementary Information. [file 41598_2022_25624_MOESM1_ESM.pdf]

Supplementary Table legends

Supplementary Table 1. Results of the real-time PCR detecting PCMV/PRV in the blood and different tissues of ten piglets of the age of nine weeks.

Supplementary Table 2. Results of the real-time PCR for PCMV/PRV in the blood and different tissues of three adult pigs (U, V, W) of the age of 41 or 43 weeks (see Table 2).

Supplementary Table 3. Comparison of two real-time PCR methods (named PCMV 1 and PCMV 2) and testing DNA from the adult pigs U, V, W using both methods.

Animal	Blood (fresh)	Blood (frozen)	PBMCs (0d, 1x10^6)	PBMCs (5d, 1x10^6)	BALF (1x10^6)	Tonsil (20 mg)	Heart (20 mg)	Bone marrow (1x10^6)
**1**	nt	nt	No Ct	No Ct	26.8	No Ct	36.0	36.0
**2**	nt	nt	No Ct	No Ct	29.4	36.8	35.2	38.8
**3**	38.3	37.1	No Ct	No Ct	30.6	No Ct	35.9	36.4
**4**	38.1	36.4	37.5	No Ct	37.5	35.9	No Ct	No Ct
**5**	36.1	38.2	36.9	38.6	No Ct	No Ct	36.9	No Ct
**6**	36.0	36.8	No Ct	No Ct	27.3	28.3	35.7	No Ct
**7**	34.7	33.4	37.9	No Ct	27.1	36.2	34.3	No Ct
**8**	36.3	35.8	38.8	No Ct	No Ct	No Ct	39.2	No Ct
**9**	37.2	No Ct	37.5	n. t.	28.1	29.1	33.4	No Ct
**10**	No Ct	No Ct	No Ct	No Ct	28.0	36.0	37.5	37.3

**1**	nt	nt	22.9	27.1	26.4	19.7	19.4	20.6
**2**	nt	nt	25.5	31.2	25.9	19.0	22.0	19.0
**3**	26.6	26.0	23.1	25.5	25.4	19.7	21.9	19.9
**4**	24.7	25.4	23.7	29.4	26.2	19.6	21.4	21.7
**5**	24.0	25.8	23.4	31.4	30.5	20.3	22.1	21.3
**6**	24.3	27.5	29.3	27.5	23.3	19.7	22.4	22.8
**7**	23.7	27.1	27.2	29.7	23.3	19.0	22.8	28.9
**8**	23.9	26.2	29.4	31.6	23.0	19.7	22.1	22.8
**9**	23.8	27.2	22.1	n. t.	23.5	19.6	22.4	18.2
**10**	24.4	26.6	27.5	31.6	22.8	20.3	22.6	18.9

Supplementary Table 1. Results in Ct values of the real-time PCR testing of 10 nine weeks old pigs (Figure 1). Upper part, results of PCMV/PRV testing, using FAM as fluorescent dye, lower part, results of the pGAPDH testing, using HEX as fluorescent dye. The Ct values are the mean value of two measurements. Sample amount was either 80 µL for blood, 1x10^6 cells for PBMCs, BALF and bone marrow and 20 mg for tonsil and heart.

PBMCs, peripheral blood mononuclear cells; 0d, tested on day 0 without incubation of the PBMCs; 5d, tested after 5 days of incubation of the PBMCs; $1x10^6$, 1 Mio cells tested; BALF, bronchoalveolar lavage fluid, 20 mg, 20 milligram tested. The DNeasy Blood & Tissue kit (Qiagen, Hilden, Germany) was used for DNA extraction. n.t., not tested; Ct, cycle threshold.

Animal	Standard procedure					Modification 1			Modification 2
	Blood (fresh)	Blood (frozen)	PBMCs (0d, 1x10^6)	PBMCs (5d, 1x10^6)	PBMCs (5d, 1x10^6)	PBMCs (0d, 3x10^6)	PBMCs (5d, 3x10^6)	PBMCs (5d, 3x10^6)	PBMCs (0d, 3x10^6)
	-	-	-	RPMI	RPMI	-	RPMI	RPMI	-
	-	-	-	-	PHA-L	-	-	PHA-L	-
	Qiagen					Qiagen			innuPREP
U	No Ct	No Ct	No Ct	No Ct	No Ct	No Ct	No Ct	No Ct	No Ct
V	No Ct	No Ct	No Ct	No Ct	No Ct	No Ct	No Ct	No Ct	No Ct
W	No Ct	No Ct	No Ct	No Ct	No Ct	No Ct	No Ct	No Ct	No Ct
U	21.1	25.8	21.9	23.8	20.6	20.3	22.1	26.0	32.8
V	22.6	25.6	22.3	23.1	23.7	20.8	22.0	21.7	29.1
W	20.9	26.1	21.5	23.0	22.0	21.0	21.3	20.3	24.2

Supplementary Table 2. Results in Ct values of the real-time PCR testing of three adult pigs (41 or 43 weeks old, see Table 1). Upper part, results of PCMV/PRV testing, using FAM as fluorescent dye, lower part, results of the pGAPDH testing, using HEX as fluorescent dye. The Ct values are the mean value of two measurements.

PBMCs, peripheral blood mononuclear cells; 0d, tested on day 0 without incubation of the PBMCs; 5d, tested after 5 days of incubation of the PBMCs; 1x10^6, 1 Mio cells tested; 3x10^6, 3 Mio cells tested; RPMI, incubation with Roswell Park Memorial Institute medium; PHA-L, activation with phytohemagglutinin-L; Qiagen, DNeasy Blood & Tissue kit (Qiagen, Hilden, Germany) used for DNA extraction; innuPREP, innuPREP Virus DNA/ RNA kit (Analytik Jena, Jena, Germany) used for DNA extraction; Ct, cycle threshold.

Sample	PCMV 1	pGAPDH	PCMV 2	pGAPDH
**10^{-7}**	No Ct	No Ct	No Ct	No Ct
**10^{-6}**	No Ct	No Ct	No Ct	No Ct
**10^{-5}**	No Ct	No Ct	No Ct	No Ct
**10^{-4}**	No Ct	34.1	No Ct	34.0
**10^{-3}**	35.9	30.6	No Ct	30.6
**10-^{2}**	33.1	27.7	36.0	26.7
**10^{-1}**	28.9	23.4	32.1	23.0
**10^{0}**	25.7	20.6	29.1	19.3
**Pig U**	No Ct	31.5	No Ct	33.3
**Pig V**	No Ct	26.9	No Ct	28.8
**Pig W**	No Ct	22.6	No Ct	24.1

Supplementary Table 3. Comparison of two real-time PCR methods (PCMV 1 and PCMV 2) using a $\log_{10}$ dilutional series of a PCMV-positive DNA sample (10^{0} until 10^{-7}) and results in Ct values of the real-time PCR testing of three adult pigs (41 or 43 weeks old, pigs U, V, W, see Table 1). In parallel the Ct values of the pGAPDH testing are shown. PCMV, porcine cytomegalovirus; Ct, cycle threshold; pGAPDH, porcine glycerinaldehyd-3-phosphat-dehydrogenase; PCR, polymerase chain reaction; DNA, desoxyribonucleic acid.

Supplementary Information file 1

Full SDS page gel, cropped in Figure 2B

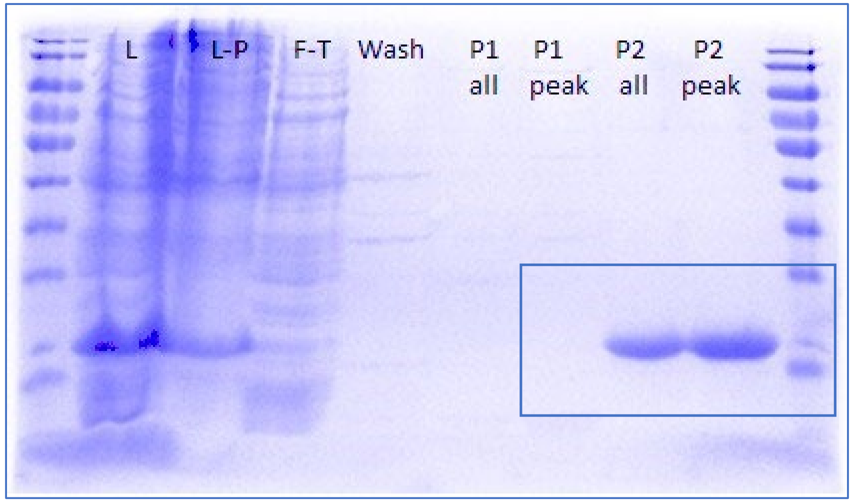

L, loaded bacterial lysate

L-P, loaded bacterial pellet

F-T, flow-through

Wash, washed material

P, peaks, see Figure 2A

Western blot, cropped in Figure 2C

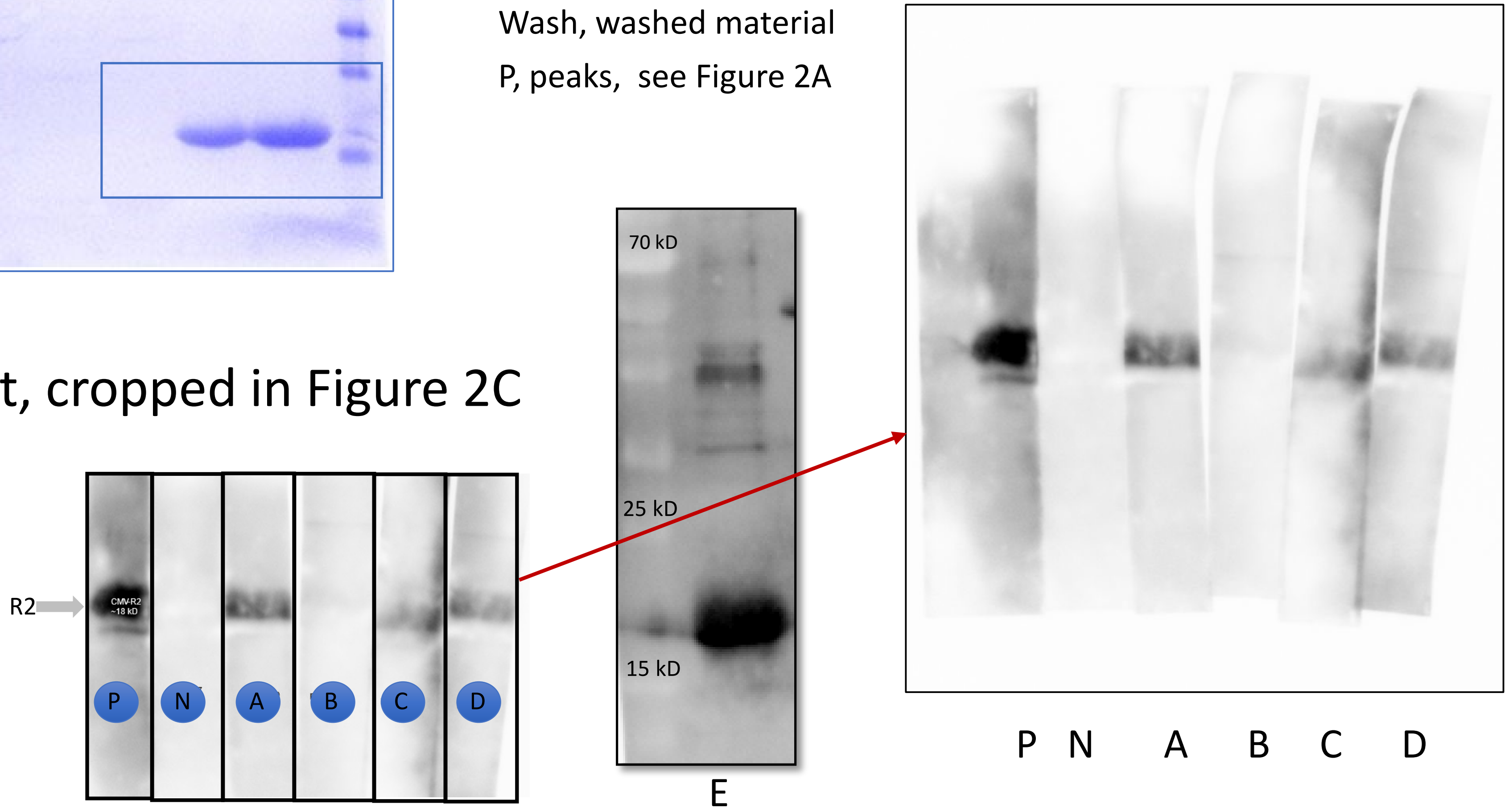

Supplementary Information file 2

Western blot, cropped in Figure 3A

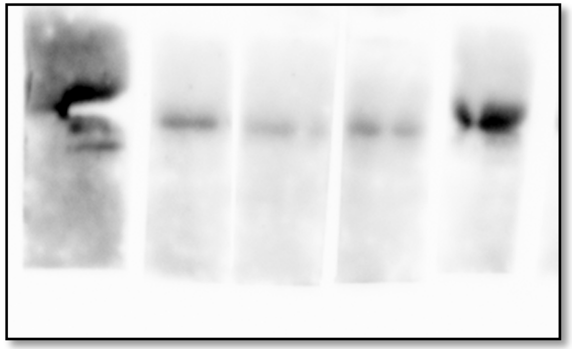

P F G H I

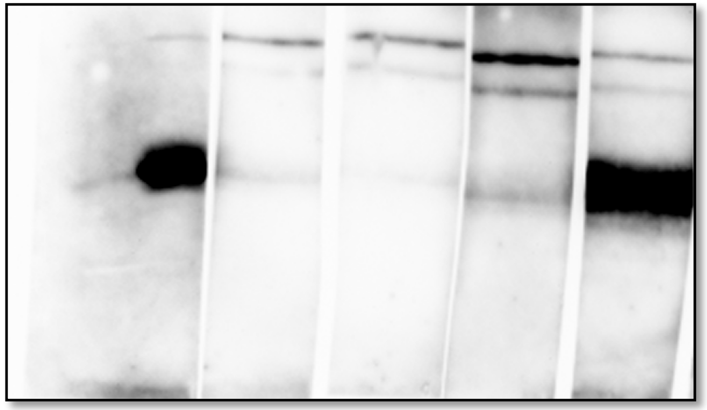

Western blot, cropped in Figure 3B, upper line

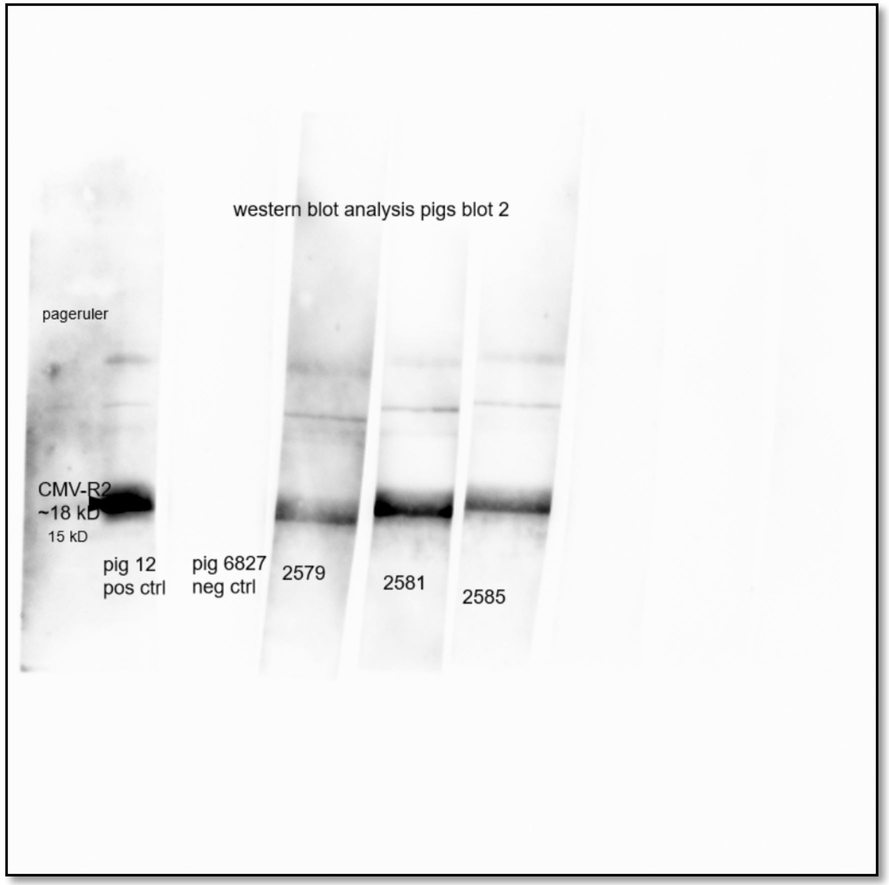

P N K L M

Supplementary Information file 3

Western blot, cropped in Figure 3B, lower line

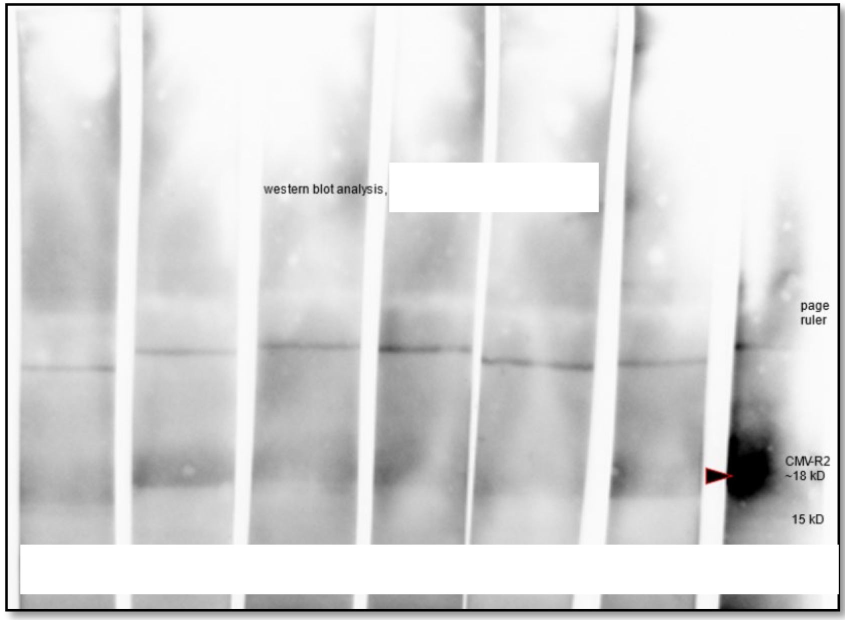

O L Q R S T P

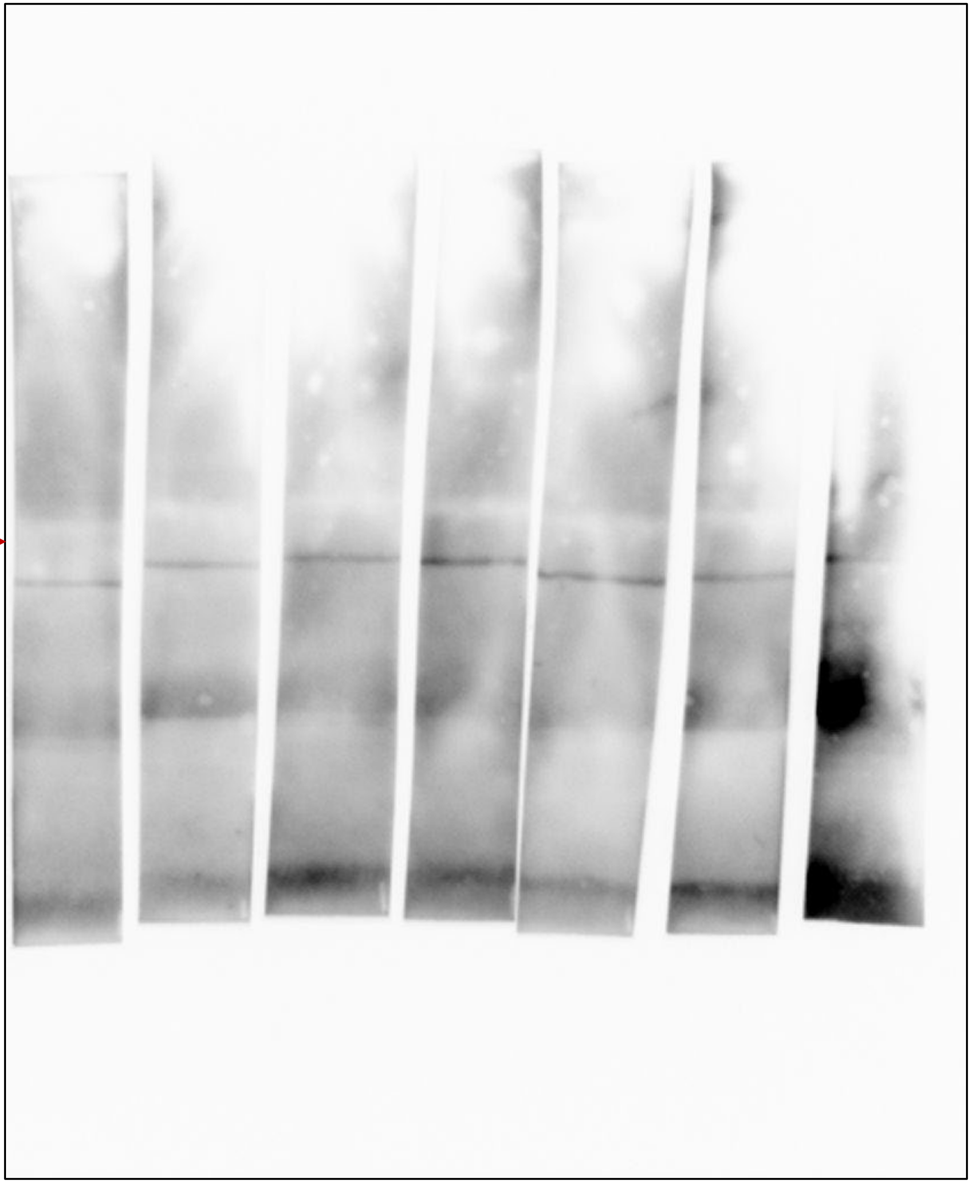

O L Q R S T P

Supplementary Information file 4

Western blot, cropped in Figure 4A

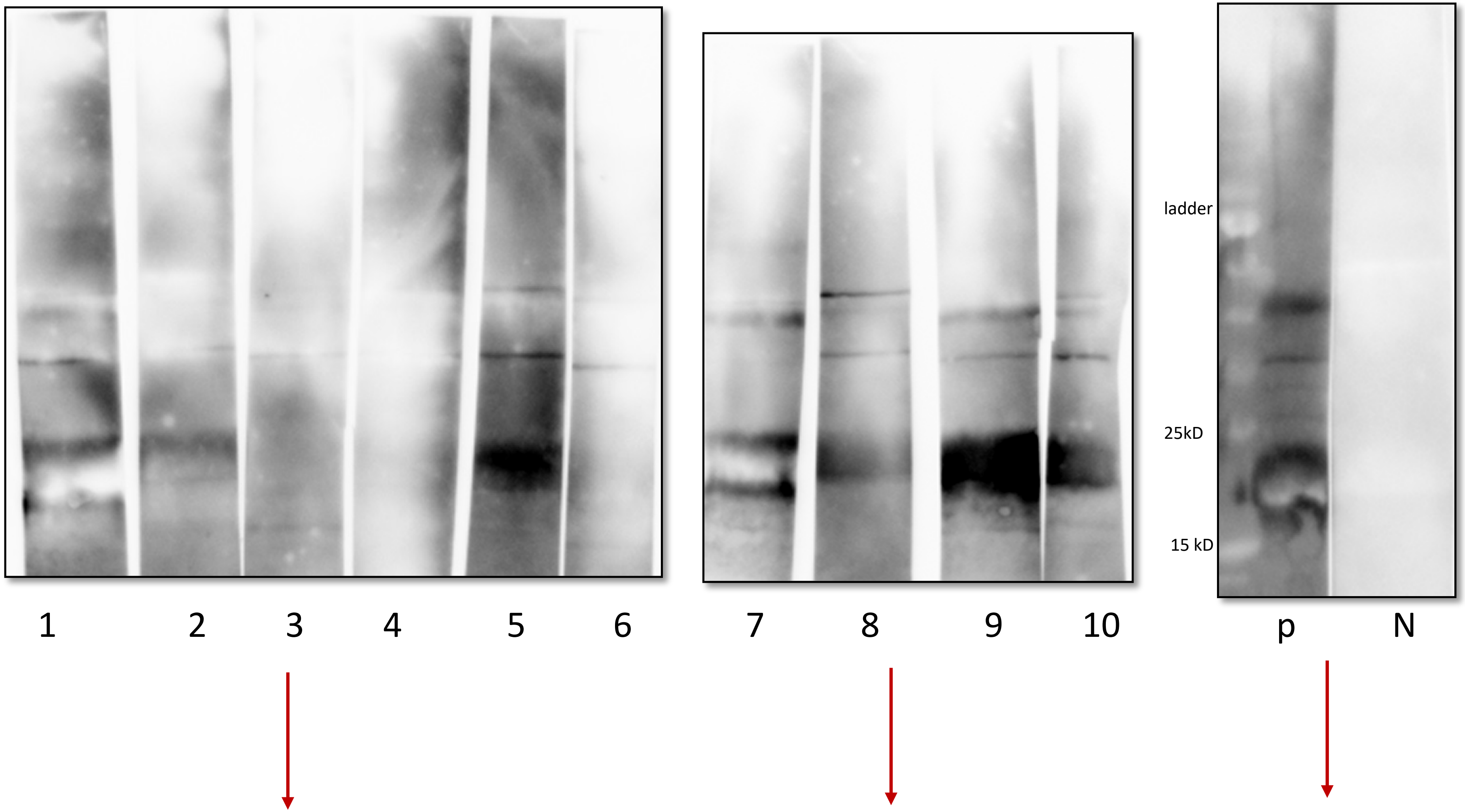

Supplementary Information file 4 (totally uncropped)

Western blot, cropped in Figure 4A

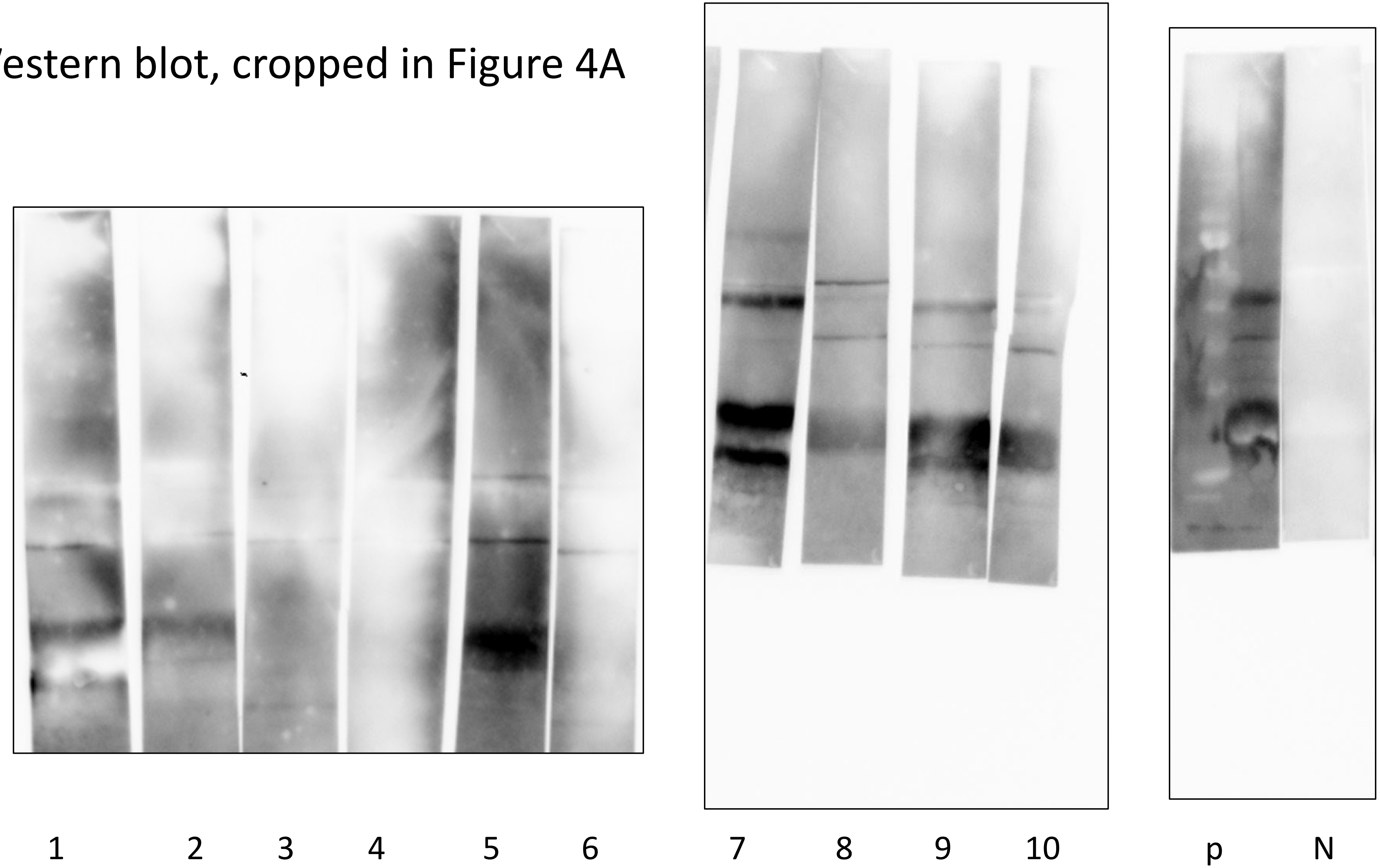

Supplementary Information file 5

Western blot, cropped in Figure 4B

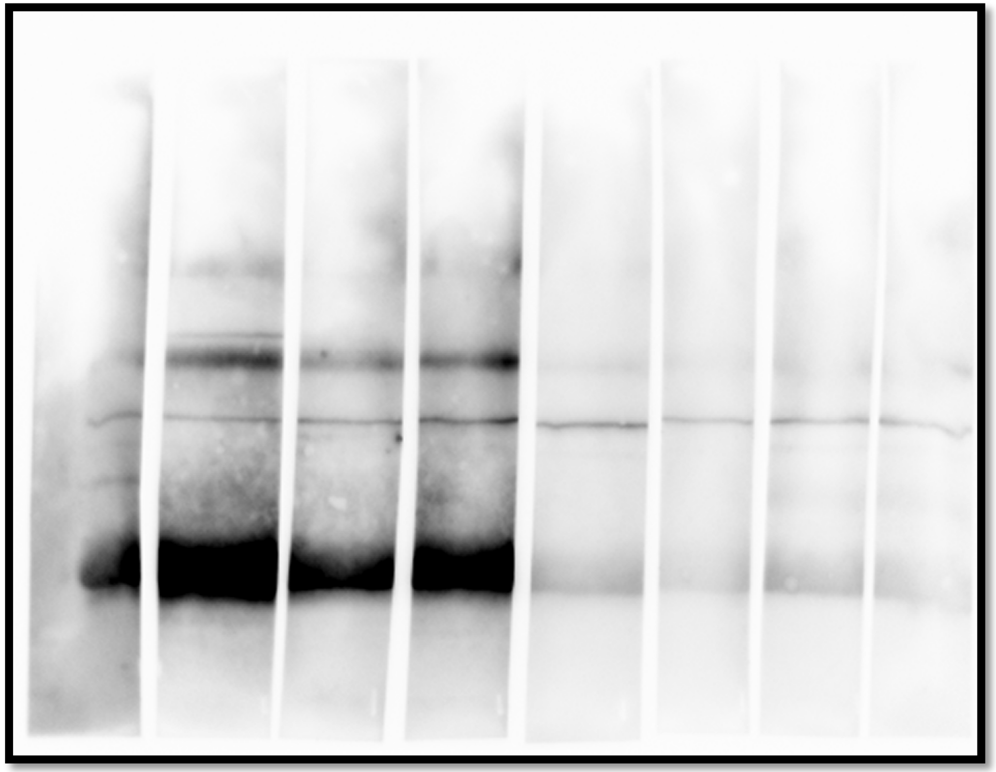

P U V W N
